# Retraction: First Report of *Vibrio tubiashii* Associated with a Massive Larval Mortality Event in a Commercial Hatchery of Scallop *Argopecten purpuratus* in Chile

**DOI:** 10.3389/fmicb.2018.02168

**Published:** 2018-08-30

**Authors:** Frontiers Editorial Office

The Journal retracts the 20 September 2016 article cited above. Following publication, the authors discovered through further analysis that the species of bacteria discovered in this study was resolved in error.

This retraction was approved by the Field Chief Editor of Frontiers in Microbiology.

